# Evaluation of Color Stability of PEEK and PEKK Dental Materials: A Systematic Review

**DOI:** 10.1002/cre2.70441

**Published:** 2026-08-26

**Authors:** Parsa Boustani, Elaheh Beyabanaki, Farnaz Taghavi‐Damghani

**Affiliations:** ^1^ School of Dentistry Shahid Beheshti University of Medical Sciences Tehran Iran; ^2^ Prosthodontics Department, School of Dentistry Shahid Beheshti University of Medical Sciences Tehran Iran

**Keywords:** color, dental materials, polyetheretherketone, polyetherketoneketone

## Abstract

**Objectives:**

Polyetheretherketone (PEEK) and polyetherketoneketone (PEKK) are two members of the polyaryletherketone (PAEK) family. PEEK is a high‐performance thermoplastic material increasingly used in dental restorations due to its favorable mechanical properties and biocompatibility. However, its long‐term clinical esthetic success significantly depends on color stability upon exposure to different coloring materials. In this systematic review, we aimed to investigate the present in vitro literature on the color stability of dental PEEK and PEKK materials when exposed to staining agents, artificial aging, and cleaning procedures.

**Materials and Methods:**

A comprehensive search in PubMed, Embase, Scopus, and MEDLINE databases was conducted up to May 15, 2026, following PRISMA guidelines. After evaluation and an updated search, 27 studies met the inclusion criteria.

**Results:**

The results indicated that PEEK generally exhibits superior color stability compared to other polymeric materials like polymethyl methacrylate (PMMA), composite resins, and polyamide. In direct comparisons, PEKK demonstrated better color stability than PEEK, attributed to its lower water sorption. Turmeric and green tea caused the most severe discoloration (Δ*E* > 3.3), while coffee, wine, and Cola produced moderate to minimal changes. Thermocycling and thermomechanical aging adversely affected PEEK, especially in ceramic‑filled variants. Surface finishing was critical—specific polishing protocols maintained color stability within clinical thresholds, whereas glazing offered no significant advantage. Cleaning methods varied in efficacy; ultrasonic and Air Flow were more effective than brushing, but sodium hypochlorite and chlorhexidine caused pronounced discoloration. While PEEK shows acceptable color stability under most conditions, it remains more susceptible to discoloration than ceramics like zirconia.

**Conclusion:**

The findings highlight the importance of material composition, surface treatment, and maintenance protocols in optimizing the esthetic performance of PEEK restorations. The limited evidence on PEKK suggests superior color stability over PEEK, warranting further investigation. Long‑term clinical studies are essential to validate these in vitro findings and to establish maintenance protocols.

## 1 Introduction

Polyetheretherketone (PEEK) and polyetherketoneketone (PEKK) are two members of the polyaryletherketone (PAEK) family (Alqurashi et al. 2021). PEEK is a high‐performance thermoplastic polymer. PEEK has excellent mechanical, thermal, and chemical resistance, as well as biocompatibility with a relatively low density (Dallal et al. 2025). PEEK also preserved satisfactory physicochemical and biological behavior under simulated oral aging conditions (Ateş and Gungormus 2026). Although PEEK was originally developed for industrial and orthopedic purposes, it has increasingly attracted attention in dentistry and is among the safest materials used to restore lost orofacial tissues (Parate et al. 2023). Its dental applications include prosthodontics, implantology, and orthodontics. As a dental restorative material, PEEK can be used to fabricate single‐unit crowns, fixed partial dentures (FPDs), and removable partial dentures (RPDs) (Parate et al. 2023). Owing to its low specific weight, PEEK can be used to construct lightweight frameworks for removable prostheses, which could provide high patient satisfaction and comfort (Bathala et al. 2019). Studies have also shown that PEEK is a suitable material for provisional restorations during implant treatment (Kwan and Kwan 2021; Zoidis, Bakiri, and Polyzois 2017, Zoidis, Bakiri, Papathanasiou et al. 2017). It is also suitable as a healing abutment (Suphangul et al. 2024). PEKK also exhibits favorable physical, mechanical, and chemical properties, making it highly suitable for a wide range of dental applications (Alqurashi et al. 2021). PEKK materials are employed as dental crowns, implants, temporary abutments, obturators, and clasps for dentures (Zol et al. 2023; Katzenbach et al. 2021).

Two fabrication methods for making PEEK and PEKK are utilized in dental restoration applications, including Computer‐Aided Design(CAD)/Computer‐Aided Manufacturing(CAM) and additive (3D‐printing) methods. PEEK and PEKK can also be combined with materials such as fibers and ceramics to improve their mechanical strength for better clinical performance (Luo et al. 2023). Milled PEEK and PEKK offer superior mechanical properties, including higher structural integrity, tensile strength, flexural strength, and fracture toughness compared to the 3D‐printed ones, making them more suitable for long‐term restorations (Limaye et al. 2022). Although 3D‐printed ones provide design flexibility and cost‐effectiveness, they may exhibit slightly lower mechanical strength compared to their milled peers. Regarding its favorable mechanical and biological characteristics, PEEK is considered a potential alternative to conventional metal and ceramic materials (Bathala et al. 2019). However, when PEEK is used in aesthetically demanding areas, its optical characteristics such as color matching and color stability should be considered for long‐term success.

In the esthetic zone, color stability is a critical factor that directly affects patient satisfaction and clinical success (Imirzalioglu et al. 2010; Samorodnitzky‐Naveh et al. 2007). Although accurate shade selection is the principal clinical consideration at the point of material selection, the long‐term durability of aesthetic restorations depends on the color stability of the chosen material (Eftekhar et al. 2023). Researchers typically employ spectrophotometry to evaluate color changes using standardized systems such as the CIELAB color system. A color difference formula (Δ*E*) is designed to give a quantitative representation of the perceived color difference between a pair of colored specimens under a given experimental condition. However, Δ*E* alone does not account for the non‐uniformity of color perception in CIELAB space. To address this limitation, more advanced formulas such as Δ*E*00 (CIEDE2000) have been developed, which incorporate weighting functions and corrections for perceptual non‐uniformities. The total color difference, expressed as Δ*E*, is calculated from L*, a*, and b* parameters obtained from a color measurement device. A higher Δ*E* value indicates a more perceptible color difference. The Δ*E* value regarded as clinically acceptable is quite different among studies (Paravina et al. 2019). Additionally, the National Bureau of Standards (NBS) index can be used to quantify and interpret the color change of a material. This index translates numerical color difference values into qualitative categories—from “very slight” to “very severe”—enabling practical and clinically relevant analysis of the results (International Organization for Standardization 2016). This quantitative approach enables the assessment of how staining agents, artificial aging, and simulated oral environments influence the color stability of PEEK and PEKK, thereby providing an objective basis for clinical decision‐making.

To determine the suitability of PEEK and PEKK in esthetic dental applications, evaluating their color stability is essential. The color stability of PEEK has been compared with other restorative materials in the literature. However, existing studies vary in methodology and findings, and no systematic review has yet summarized the evidence regarding the color stability of PEEK under different staining and aging conditions. This systematic review aimed to summarize available in vitro studies on the color stability of dental PEEK materials, identify the main factors contributing to discoloration, compare its performance with other restorative materials, and highlight research gaps for future studies. Due to the limited number of studies on PEKK, only a brief, non‐systematic discussion of its color stability is provided based on the existing literature.

## 2 Materials and Methods

This systematic review was done according to the Preferred Reporting Items for Systematic Reviews and Meta‐Analyses (PRISMA) statement (Page et al. 2021) and was prospectively registered in the PROSPERO database (Registration number: CRD420251031097).

### 2.1 Eligibility Criteria

#### 2.1.1 Population

In vitro specimens of dental PEEK and PEKK materials, including unfilled, ceramic‑filled, and pigmented variants.

#### 2.1.2 Intervention

Exposure to staining agents (e.g., coffee, tea, turmeric, green tea, Cola, red wine, mouthrinses, cigarette smoke), artificial aging (thermocycling, thermomechanical aging), and cleaning/maintenance procedures (mechanical brushing, denture cleansers, disinfectants).

#### 2.1.3 Comparison

Other dental restorative materials such as polymethyl methacrylate (PMMA), composite resins, polyamide, acetal resin (POM), zirconia, lithium disilicate, hybrid ceramics, and PEKK. Different surface treatments (polishing vs. glazing) and material compositions (filled vs. unfilled PEEK) were also compared.

#### 2.1.4 Outcome

Color stability measured as color change (Δ*E* or Δ*E*00) using spectrophotometry, assessed against clinical acceptability thresholds or National Bureau of Standards (NBS) units.

The Inclusion Criteria were as follows:

1. In vitro studies investigating color stability of PEEK or PEKK materials.
2. In vitro studies including artificial staining procedures by liquids, mouthrinses, and smoking.
3. In vitro studies analyzing color stability only with water aging/thermocycling procedures.
4. In vitro studies that measured color changes using color‐difference formulas.
5. Publications in English.

Exclusion Criteria included:

1. Clinical trials, case reports, reviews, or animal studies.

### 2.2 Information Sources

A literature search was conducted by two independent reviewers for English‐language papers published in dental journals up to August 12, 2025. Later, the search was updated to May 15, 2026. The search was done in the following databases: PubMed, Embase, Scopus, and Medline. Manual searching was done in Google Scholar for possibly additional records.

### 2.3 Search Strategy

The database‐specific queries are presented in Table 1.

**Table 1:** The complete search strategies and database‐specific queries used in the systematic literature review.

Data base	Search query
Embase	(‘coloring agent’/exp OR ‘coloring agent’ OR ‘color stability’/exp OR ‘color stability’ OR ‘stain’/exp OR ‘stain’ OR ‘stain susceptibility’ OR ‘staining’/exp OR ‘staining’ OR ‘discoloration’/exp OR ‘discoloration’) AND (‘PEEK’/exp OR ‘PEEK’ OR ‘PEKK’ OR ‘polyetheretherketone’/exp OR ‘polyetheretherketone’ OR ‘polyetherketoneketone’/exp OR ‘polyetherketoneketone’)
Medline	((TI = (((PEEK OR PEKK OR polyetherketoneketone OR polyetheretherketone)) AND ((coloring agent) OR (color stability) OR (stain) OR (stain susceptibility) OR (staining) OR (discoloration)))) OR (TS = (((PEEK OR PEKK OR polyetherketoneketone OR polyetheretherketone)) AND ((coloring agent) OR (color stability) OR (stain) OR (stain susceptibility) OR (staining) OR (discoloration)))) OR (AB = (((PEEK OR PEKK OR polyetherketoneketone OR polyetheretherketone)) AND ((coloring agent) OR (color stability) OR (stain) OR (stain susceptibility) OR (staining) OR (discoloration))))
Scopus	TITLE‐ABS‐KEY(((“coloring agent”) OR (“color stability”) OR (“stain”) OR (“stain susceptibility”) OR (“staining”) OR (“discoloration”)) AND ((“PEEK”) OR (“PEKK”) OR (“polyetheretherketone”) OR (“polyetherketoneketone”)))
PubMed	((coloring agent) OR (color stability) OR (stain) OR (stain susceptibility) OR (staining) OR (discoloration)) AND ((PEEK) OR (PEKK) OR (polyetheretherketone) OR (polyetherketoneketone))

### 2.4 Study Selection

Two authors independently reviewed the titles and abstracts according to the predefined eligibility criteria. Then full‐text screening was performed, and final included articles were determined through consensus. Disagreements over the selection of articles were reviewed and resolved by the third author.

### 2.5 Data Extraction

The data items that were collected include: type of study, study objective, PEEK and PEKK material used, other restorative materials tested, cleansing materials, staining medium, other processes applied, test duration, spectrophotometer model, finishing/polishing procedure, Δ*E* threshold, and study outcomes.

### 2.6 Risk of Bias Assessment

For assessing the risk of bias, we used the QUIN (Quality Assessment Tool for In Vitro Studies) tool (Sheth et al. 2024). The final assessment was performed by grading each study as “low,” “medium,” or “high” risk of bias. Two reviewers independently conducted the risk of bias assessment. Any disagreements were first discussed to reach consensus; if consensus could not be achieved, a third reviewer was consulted for final decision.

## 3 Results

### 3.1 Study Selection

**Figure 1:**
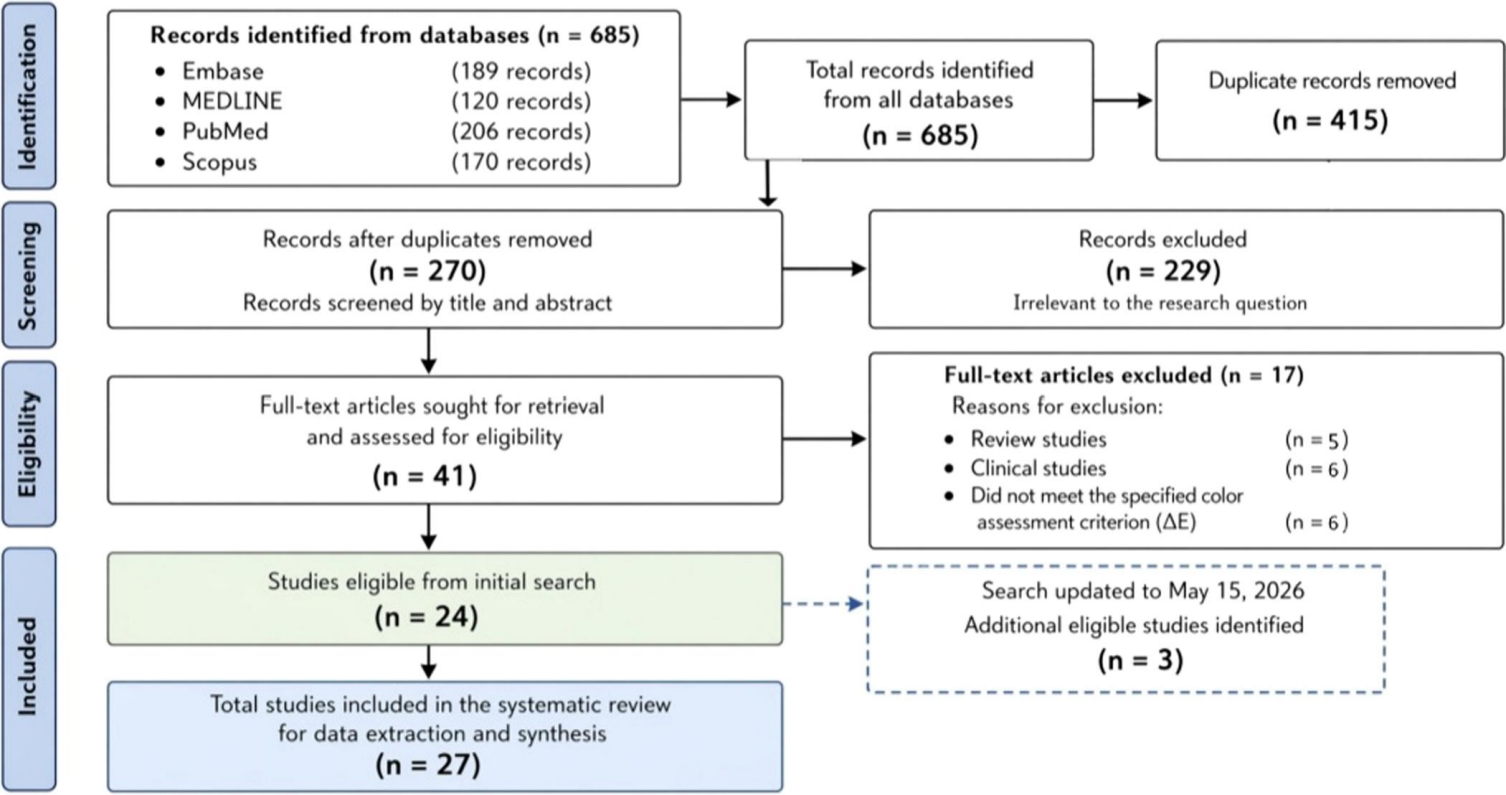
PRISMA flow diagram of study selection.

The systematic search of electronic databases identified a total of 685 records (Embase: 189; MEDLINE: 120; PubMed: 206; Scopus: 170). After importing these records into reference management software (EndNote 20, Clarivate Analytics, Philadelphia, PA, USA) and removing duplicates, 270 records remained for initial screening. In the first screening phase, the titles and abstracts of these 270 records were reviewed, leading to the exclusion of 229 records due to the title/abstract being irrelevant to the research question. Consequently, 41 full‐text articles were assessed for eligibility. After a full‐text review of these 41 articles, 17 articles were excluded for the following reasons: five were review studies, six were clinical studies, and six articles did not meet the specified color assessment criterion (Δ*E*). The study selection process was summarized in the PRISMA flow diagram (Figure 1). The literature search was updated to May 15, 2026, which yielded three additional studies (Alharthi et al. 2025; Kar et al. 2025; Ateş and Gungormus 2026) that fully met the eligibility criteria and were consequently included. Finally, 27 studies (Ateş and Gungormus 2026; Demir Sevinç et al. 2024; Tekin 2021; Shiozawa et al. 2023; Porojan et al. 2021, 2023; Diken Türksayar et al. 2023; Heimer et al. 2017; Porojan et al. 2022; Abhay et al. 2021; Saraswathy et al. 2024; Makkeyah et al. 2024; Perween et al. 2025; Kamal 2020; Cinel Sahin and Mutlu Sagesen 2024; Erdağ et al. 2023; Kaya et al. 2024; Ozyilmaz et al. 2021; Pattarakitniran et al. 2024; Singh et al. 2024; Chidembaranathan et al. 2024; Ahmed and Rattan R 2023; Papathanasiou et al. 2022; Polychronakis et al. 2020; Sultan and Salloum 2024; Alharthi et al. 2025; Kar et al. 2025) that met the inclusion criteria for this systematic review were selected for data extraction and synthesis.

### 3.2 Risk of Bias Assessment

Based on the QUIN tool, out of the total 27 studies included, 7 studies (25.9%) were categorized as “Low” risk of bias and 20 studies (74.1%) as “Moderate” risk of bias. No study was classified as “High” risk. The overall quality scores ranged from 58.3% to 77.2%. Table 2 presents the detailed risk of bias assessment scores for each included study.

**Table 2:** Quality assessment (QUIN) scores for each included study.

First author, year	Criteria 1	Criteria 2	Criteria 3	Criteria 4	Criteria 5	Criteria 6	Criteria 7	Criteria 8	Criteria 9	Criteria 10	Criteria 11	Criteria 12	Total score	Final score %	Risk of bias
Demir Sevinç et al. (2024)	2	0	2	2	2	0	1	2	0	0	2	2	15	62.5	Medium
Makkeyah et al. (2024)	2	0	2	2	2	0	0	2	0	0	2	2	14	58.3	Medium
Porojan et al. (2021)	2	0	2	2	2	0	1	2	0	0	2	2	15	62.5	Medium
Porojan et al. (2023)	2	0	2	2	2	0	0	2	0	0	2	2	14	58.3	Medium
Shiozawa et al. (2023)	2	0	2	2	2	0	0	2	0	0	2	2	14	58.3	Medium
Perween et al. (2025)	2	2	2	2	2	0	1	2	0	0	2	2	17	70.83	Low
Polychronakis et al. (2020)	2	2	2	2	2	0	1	2	1	0	2	2	18	75	Low
Sultan and Salloum (2024)	2	0	1	2	2	0	N/A	2	0	0	2	2	13	59	Medium
Abhay et al. (2021)	2	2	2	2	2	0	0	2	0	0	2	2	16	66.6	Medium
Heimer et al. (2017)	2	0	2	2	2	1	1	2	0	0	2	2	16	66.6	Medium
Diken Türksayar et al. (2023)	2	2	2	2	2	0	N/A	2	1	0	2	2	17	77.2	Low
Saraswathy et al. (2024)	2	0	2	2	2	0	0	2	0	0	2	2	14	58.3	Medium
Papathanasiou et al. (2022)	2	2	2	2	2	0	1	2	0	0	2	2	17	70.83	Low
Ahmed and Rattan (2023)	2	2	2	2	2	1	1	2	0	0	2	2	18	75	Low
Porojan et al. (2022)	2	0	2	2	2	0	N/A	2	0	0	2	2	14	63.6	Medium
Kamal (2020)	2	0	2	2	2	0	0	2	0	0	2	2	14	58.3	Medium
Kaya et al. (2024)	2	2	2	2	2	0	0	2	0	0	2	2	16	66.6	Medium
Ozyilmaz et al. (2021)	2	0	2	2	2	1	1	2	0	0	2	2	16	66.6	Medium
Pattarakitniran et al. (2024)	2	0	2	2	2	1	1	2	0	0	2	2	16	66.6	Medium
Singh et al. (2024)	2	2	2	2	2	0	N/A	2	0	0	2	2	16	72.7	Low
Sai Madhuri Nemani (2024)	2	2	2	2	2	0	0	2	0	0	2	2	16	66.6	Medium
Tekin (2021)	2	0	2	2	2	0	1	2	0	0	2	2	15	62.5	Medium
Cinel Sahin and Mutlu Sagesen (2024)	2	2	2	2	2	1	0	2	1	0	2	2	18	75	Low
Erdağ et al. (2023)	2	0	2	2	2	0	1	2	0	0	2	2	15	62.5	Medium
Kar et al. (2025)	2	0	2	2	2	0	1	2	0	0	2	2	15	62.5	Medium
Alharthi et al. (2025)	2	0	2	2	2	0	1	2	0	0	2	2	15	62.5	Medium
Ateş and Gungormus (2026)	2	2	2	2	2	0	0	2	0	0	2	2	16	66.6	Medium

*Note:* The criteria are numbered as follows: 1. Clearly stated aims/objectives. 2. Detailed explanation of sample size calculation. 3. Detailed explanation of sampling technique. 4. Details of comparison group. 5. Detailed explanation of methodology. 6. Operator details. 7. Randomization. 8. Method of measurement of outcome. 9. Outcome assessor details. 10. Blinding. 11. Statistical analysis. 12. Presentation of results.

### 3.3 Descriptive Analysis of the Included Studies

The extracted data and methodological details of the included studies are presented in Tables 3 and 4.

**Table 3:** Characterization of restorative materials (PEEK/PEKK and comparative groups), study objectives, clinical acceptability thresholds (Δ*E*), and final color stability outcomes.

First author, year	Objective	PEEK or PEKK material	Other restorative materials in test	Δ*E* Clinical acceptability threshold used in article	Outcomes
Demir Sevinç et al. (2024)	To investigate color stability and surface roughness of polyetheretherketone (PEEK), zirconia, and hybrid ceramics while stored in different liquids.	Copra PEEK (PEEK), Whitepeaks, Germany, 100% PEEK	Zirconia, Incoris TZI (ZR), Dentsply Sirona, Germany, ZrO_2_ + HfO_2_ + Y_2_O_3_, 99% by weight. Hybrid resin nanoceramic, Cerasmart GC (HC), GC Dental Product Corp. Japan, 71% nanoparticle filling includes silica and barium glass	3.3	The Δ*E* values of the HC specimens were mostly clinically acceptable and were significantly lower than ZR and PEEK specimens on the 6th and 12th days (*p* < 0.05). The 12th‐day Δ*E* values of PEEK specimens were higher than the acceptable threshold in nearly all solutions, whereas the ZR specimens’ Δ*E* values were very close to the clinically acceptable threshold.
Fatma Makkeyah et al. (2024)	To determine the effects of conventional cigarette smoking (CS) and recent heated tobacco products (HTPs) on the surface roughness and color stability of different indirect restorative materials.	CAD/CAM polyetheretherketone (BioHPP bredent GmbH, Germany)	CAD/CAM lithium disilicate glass–ceramic (IPS e.max CAD; Ivoclar Vivadent, Liechtenstein), CAD/CAM zirconia (BruxZir Zirconia, Glidewell, USA)	3.3	The color change of the samples was affected both by the type of smoking and the material. HTP caused a significantly smaller change in color than cigarette smoking, zirconia caused a significantly smaller change in color than did PEEK, and lithium disilicate caused a significantly smaller change in color than did PEEK.
Porojan et al. (2021)	To investigate the long‐term effect of the combined action of aging and immersing solutions on the optical properties and color stability of PEEK material, related to surface processing (polishing or glazing).	20% ceramic fillers in a high‐performance polymer (bioHPP, Bredent, Senden, Germany)	_	To relate the color change to a clinical standard, the E* values were converted into NBS units: NBS = E* 0.92	It has been demonstrated that the polished and glazed samples had no statistically significant difference in the overall color change after undergoing aging and coffee exposure (*p* = 0.061). Artificial aging has a negative effect on color stability for all samples. Only samples thermally cycled in hot coffee registered perceivable color changes.
Porojan et al. (2023)	To evaluate in vitro, the surface roughness development and color changes related to staining beverages and cleaning methods after simulated periods of clinical use.	Finoframe PEEK A2 (F) (Fino GmbH, Bad Bocklet, Germany); Juvora medical PEEK nature (J) (Juvora Ltd. Global Technology Center, Lancashire, UK), both 100% Polyetheretherketone (PEEK); BioHPP dentine shade 2 (B) (Bredent group GmbH & Co.KG, Senden, Germany), a ceramic reinforced partially crystalline polyetheretherketone (PEEK)	_	Not mentioned	Color changes were perceivable for J in coffee and B in tea. Brushing eliminated all perceivable discoloration. Chemical cleaning removed stains from tea and coffee but not from juice for J. J showed the lowest color stability, especially in juice. F remained mostly color stable across all conditions.
Shiozawa et al. (2023)	To clarify the discoloration of fiber‐reinforced composite resin (FRC) disc materials.	Polyetheretherketone, JUVORA dental disc, Juvora	FRC: Fiber‐reinforced composite resin, TRINIA, Shofu, glass fiber, epoxy resin HCR: Composite resin, Disk HC, Shofu, UDMA, TEGDMA, silica powder, micro fumed silica, zirconium silicate	2.7	HCR exhibited the greatest Δ*E* values, followed by FRC and PEEK during this period, and the coffee and curry solutions exhibited similar tendencies.
Perween et al. (2025)	To understand the color retention capabilities of dental materials polyetheretherketone (PEEK), composite, and acrylic resin under the influence of different staining substances found in Indian food and beverages, using spectrophotometric analysis.	Polyetheretherketone (Poly Fluoro Ltd.)	Composite (Brilliant NG, Coltene), and self‐cure acrylic resin (Tooth molding material, DPI)	3.7	PEEK samples exhibited the highest color stability, whereas composite samples were the least color stable. Turmeric and coffee had the highest staining impact on all materials, while cola and distilled water showed minimal effect. All test samples in turmeric solution exhibited clinically unacceptable color change. PEEK samples in distilled water, coffee, and cola demonstrated color change that can be clinically acceptable.
Polychronakis et al. (2020)	To investigate the long‐term effect of staining and/or cleansing solutions on the color stability of two non‐metal removable partial denture materials.	BioHPP, Ceramic filled (20%) Polyetheretherketone, Bredent, GmbH & Co. KG, Senden, Germany	TSM Acetal Dental, Polyoxymethylene (POM), Pressing Dental Srl, San Marino, Italy	1.87	POM material showed a much higher (4 fold) discoloration than PEEK in coffee, a little lower in wine, and the same in combo (coffee + cleanser), cleanser, and control. With the exception of POM in coffee, all other values were within acceptability thresholds.
Sultan and Salloum (2024)	To compare the color stability of different denture base materials through an in vitro approach.	CopraPeek, Light, Polyetheretherketone(≈ 80%) Titanium dioxide (< 20%) Other additives (< 0.1%)	CAD/CAM polymethyl methacrylate (PMMA) (Vita Vionic, Vita) and CAD/CAM Acetal (AC) (Zirlux Acetal, Glidewell)	Not mentioned	PEEK exhibited superior color stability compared to PMMA and AC denture base materials under artificial aging conditions.
Abhay et al. (2021)	To compare the wear resistance, abrasiveness, color stability, and displacement resistance of zirconia and PEEK milled crowns.	PEEK (BioHpp, Bredent GmbH, Senden, Germany)	Zirconia (CaroZiir S, Carol Zircolite Pvt. Ltd., Gujarat, India)	Not mentioned	The values of color stability significantly decreased (*p* < 0.001) when the zirconia and PEEK crowns underwent wear and aging in the chewing simulator. The color stability of thermocycled and worn zirconia was significantly better than that of the PEEK crowns. PEEK crowns still showed good color stability.
Heimer et al. (2017)	To assess the discoloration and stain removal potential of different cleaning methods relevant to individual/professional prophylaxis and laboratory cleaning on polyetheretherketone (PEEK), poly(methyl methacrylate) (PMMA)‐based, and composite (COMP) materials after storage in different media for 7 days.	Ceramic‐filled (20%) PEEK, bioHPP, bredent, Senden, Germany	99% PMMA polymer (bredent, Senden, Germany), Bis‐GMA composite with microfillers (bredent, Senden, Germany)	3.3	PEEK showed the significantly (*p* < 0.001) lowest discoloration after a 1‐week storage over all coloring media as compared to PMMA and COMP.
Diken Türksayar et al. (2023)	To evaluate the surface roughness and color stability of novel monolithic CAD‐CAM materials after thermomechanical aging	Polyetheretherketone, ceramic filler (20%) (BioHPP‐Bredent)	IPS e.max CAD; Ivoclar Vivadent (EX), Cerasmart; GC Corporation (RC), Brilliant Crios; Coltène (CO)	1.8	Ceramic‐reinforced PEEK presented the greatest color difference after thermomechanical aging, and among the materials tested, only the color change of ceramic‐reinforced PEEK was greater than the clinically acceptable threshold.
Saraswathy et al. (2024)	To compare the surface roughness and color stability of polyetheretherketone (PEEK) with those of conventional interim prosthetic materials like polymethylmethacrylate, bis‐acrylic composite, and rubberized diurethane dimethacrylate, following immersion in solutions of varying pH value.	Gp P: Polyetheretherketone, BioHPP, Bredent, Chesterfield, Derbyshire, UK	Gp R: Rubberized diurethane dimethacrylate, Tuff‐temp Plus, Pulpdent Corp., Watertown, Massachusetts, USA Gp B: Bis‐acrylic composite, Protemp 4, 3M India Ltd., Bengaluru, Karnataka, India Gp A: Autopolymerized polymethylmethacrylate, Semident SC‐10, Samit Dental Products, Jhandewalan, New Delhi, India	Not mentioned	Polyetheretherketone was the most color stable interim material in all solutions, except green tea. Green tea showed the maximum staining potential among the tested solutions for all interim materials except PMMA.
Papathanasiou et al. (2022)	To assess the effect of commonly used solutions on color stability, gloss, and surface roughness of removable partial dental prostheses polymers.	BioHPP, Modified PEEK, PEEK: 80%, ceramic fillers: 20%, Bredent GmbH & Co.KG	A polyamide (Valplast; Valplast Int Corp), an acetal resin (T.S.M. Acetal; PRESSING DENTAL Srl, San Marino, San Marino) and a heat‐polymerized acrylic resin (Paladon 65; Kulzer GmbH) that was used as the control	3.3	Polyamide presented the highest color change overall (ΔΕ* = 14.59 ± 8.65) while PEEK had the lowest (ΔΕ* = 3.83 ± 2) (*p* < 0.001). With regard to the stability of color, gloss, and surface roughness, PEEK could be a promising material for RPD fabrication.
Ahmed and Rattan (2023)	To assess and compare the color stability of PEEK, polyan, and biodentaplast denture base materials (DBMs) upon staining with distilled water, tea, coffee, and turmeric solutions after 1 day, 7 days, and 30 days.	PEEK	Polyan, biodentaplast	3.3	PEEK has a higher color stability in comparison with modified PMMA materials.
Porojan et al. (2022)	To evaluate the surface characteristics, microhardness, and color stability of PEEK materials related to water saturation and in vitro aging.	BioHPP1. Dentine shade 2, Ceramic reinforced (grain size of 0.3 to 0.5 m) partially crystalline polyetheretherketone (PEEK), Bredent group GmbH & Co. KG, Senden, Germany (B), 2. Finoframe PEEK A2(F), Polyetheretherketone (PEEK) 100%, Fino GmbH, Bad Bocklet, Germany, 3. Juvora Medical PEEK nature (J), Polyetheretherketone (PEEK) 100%, Juvora Ltd. Global Technology Center, Lancashire, UK	_	To relate the color change to a clinical standard, the E* values were converted into NBS units: NBS = E* 0.92	In relation to the levels of color change, according to the NBS, after 4 weeks of water immersion, they are between extremely slight and slight for all materials. Thermocycling induces extremely slight changes for B and J and slight for F. B is more changed by immersion.
Kamal (2020)	To compare the color stability of Polyetheretherketone (PEEK), Acetal resin (polyoxymethylene POM), and Acrylic resin (Polymethylmethacrylate PMMA) denture base material discs milled by CAD/CAM and stored in different storage media.	CAD/CAM PEEK (Bredent, Germany)	CAD/CAM Acetal resin (Bredent, Germany) and CAD/CAM acrylic resin blanks (Ivoclar Vivadent Inc. USA)	5.6	The least color changes were represented by Acetal resin denture base material, followed by PEEK denture base material, while the highest color changes were represented by Acrylic resin denture base material. Coffee solution caused the higher discoloration values compared to ginger solution. PEEK material showed clinically unaccepted values only with coffee solution.
Kaya et al. (2024)	To assess color stability, solubility, and water sorption on polyether ether ketone (PEEK) and polyether ketone ketone (PEKK) after immersion in different storage conditions.	PEEK (CopraPeek Whitepeaks Dental Solutions GmbH & Co. KG, Wesel, Germany) (A2 light), PEKK (Pekkton Ivory Cendres + Metaux SA, Switzerland)	—	2.25	Color change and water sorption values were higher in the PEEK samples than in the PEKK samples. While PEEK showed color change exceeding the acceptable level in all samples, in PEKK, the color change was under the acceptability threshold. In PEEK and PEKK CAD/CAM blocks, samples kept in coffee showed the highest color change values, followed by red wine and distilled water, in that order.
Ozyilmaz et al. (2021)	To evaluate the effect of sodium perborate effervescent tablets and citric acid solution on the color stability and surface topography of a new generation of high‐performance polymer polyetherketoneketone (PEKK), thermoinjection‐molded polyamide, and polymethylmethacrylate (PMMA).	PEKK high performance polymer (Polyetherketoneketones, titanium dioxide pigments), Cendres + Metaux, Biel, Switzerland (P group)	Deflex polyamide/nylon (99.6% polyamide−0.4% allowed pigments), Nuxen SRL, Buenos Aires, Argentina(D group), Meliodent heat‐polymerized acrylic resin (Polymethylmethacrylate, benzoylperoxide, methyl methacrylate, ethylene glycol dimethacrylate), Heraeus Kulzer, Hanau, Germany (M group)	3.7	The Perlodent denture cleanser demonstrated the highest Δ*E* value in all groups. This difference was observed to be statistically significant in the P group (*p* < 0.05) compared with other denture cleansers. However, in the M and D groups, Perlodent, Protefix, and Corega had no statistically different Δ*E* value (*p* > 0.05). For patients who have PEKK and polyamide‐based prosthesis, the use of citric acid‐based cleansers may be more recommended than sodium perborate‐containing cleansers because of its clinically acceptable color changes on polymers in terms of color stability.
Pattarakitniran et al. (2024)	To compare the color change and surface roughness of polyetheretherketones (PEEK) immersed in various beverages and mouthrinses for 7 days.	Cylindrical PEEK blanks provided by the manufacturer (Smile PEEK, Pressing Dental SRL, San Marino, Italy)	_	3.3	Povidone‐iodine caused clinically unacceptable discoloration, while red wine led to a slight but clinically acceptable change. Other solutions had minimal impact on PEEK's color.
Singh et al. (2024)	This study investigates the interaction of zirconia and polyetheretherketone (PEEK) with indirect composite in fixed dental prostheses. This investigation aimed to assess the shear bond strength (SBS) and color stability of zirconia and PEEK before and after aging, addressing critical concerns in dental restorative applications.	CAD/CAM milled PEEK, polyetheretherketone, Intamsys PEEK, Intamsys, Shanghai, China Shenzhen Upcera Dental	Indirect composite resin (IC), Shofu Ceramage, 53 vol% of 1 µ SiO_2_ and Al_2_O_3_ inorganic fillers + matrix‑25 wt% co‑polymers + 22% conventional resins, Shofu Dental, India CAD/CAM milled zirconia (Zr), ST Multilayer Zirconia, ZrO_2_ > 97.7%+ HfO_2_ + Y_2_O_3_ 4.4%–5.5% Y_2_O_3_ < 0.5% Al_2_O_3_ < 0.3% Fe_2_O_3_ < 1.0% Er_2_O_3_ and other oxides < 1.2%, Technology Co. Ltd., Guangdong, China	Not mentioned	Indirect composite layered with PEEK demonstrated superior color stability compared to indirect composite layered with zirconia.
Sai Madhuri Nemani (2024)	To evaluate the color stability and surface roughness of PEEK after immersion in 10% povidone‐iodine, 4% chlorhexidine gluconate, 2% glutaraldehyde, 5% sodium hypochlorite, and neutral soap disinfectant solutions.	CAD/CAM PEEK (BioHPP, Bredent, Senden, Germany)	_	Not mentioned	The samples immersed in sodium hypochlorite showed the most remarkable color change, followed by neutral soap, chlorhexidine, and povidone‐iodine, with glutaraldehyde.
Tekin (2021)	Compare the long‐term effect of overnight use of denture cleansers with different chemical compositions on the color stability of denture base polymers	Unfilled PEEK CAD/CAM disc‐ Juvora Dental Disc; Juvora, London, UK	Thermoinjection‐molded polyamide (PA group), auto‐polymerized polymethylmethacrylate (PMMA) (AP group), and heat‐polymerized resin PMMA (HP group)	4.08	PEEK is more susceptible to color changes than AP and HP, especially with Corega tablet, where it shows the highest Δ*E*00 value, but it performs better than PA in Protefix tablet, 0.5% NaOCl solution, and distilled water.
Cinel Sahin and Mutlu Sagesen (2024)	To evaluate the effects of different polishing protocols on the surface properties and color stability of the polyetheretherketone (PEEK).	CopraPeek Light, Polyetheretherketone (≈ 80%) Titanium dioxide (< 20%) Other additives (< 0.1%), Whitepeaks Dental Solutions GmbH & Co. KG, Germany	_	1.8	∆*E*00 measurements of the Abraso–Starglanz polishing paste group and Enhance polishing system group were found to be below the acceptability threshold value. The values obtained for all other polishing protocols were above the threshold limit.
Erdağ et al. (2023)	The effect of different denture cleaning methods on the surface roughness and color stability of polyether ether ketone (PEEK) dental materials was studied	CAD/CAM milled PEEK discs (CopraPeek, Whitepeaks Dental Solutions, Germany)	_	1.8	No statistically significant difference was found between control and other test groups, in terms of Δ*E*00 values. All Δ*E*00 values were clinically acceptable. Considering the color stability, many cleaning procedures can be used safely for PEEK polymers used for dental applications.
Kar et al. (2025)	To investigate the impact of various mouthrinses on the color stability of IPS e.max CAD, Zirconia CAD and PEEK CAD blocks.	PEEK	IPS e.max CAD (lithium disilicate), Zirconia CAD (yttria‑stabilized zirconia)	To relate the color change to a clinical standard, the E* values were converted into NBS units: NBS = E* 0.92	IPS e.max showed the highest color stability (lowest Δ*E*), followed by zirconia, then PEEK. Listerine and chlorhexidine caused the most pronounced color changes across all materials. Herbal mouthwash had a lesser impact. Distilled water produced the least change. All Δ*E* differences were statistically significant (*p* < 0.05) except in the control group.
Alharthi et al. (2025)	To evaluate and compare the effects of thermal aging (thermocycling) on the color stability and mechanical properties of three CAD/CAM polymers used for long‑term provisional restorations.	PEEK (BioHPP, Bredent GmbH) – polyetheretherketone with 20 wt% titanium dioxide ceramic filler and aluminum oxide sand.	CAD‑Temp (VITA Zahnfabrik) – PMMA (83–86 wt%) with silica microfillers; Everest C‑Temp (KaVo) – high‑performance glass fiber‑reinforced polymer.	3.3	PEEK showed the lowest color change (Δ*E* = 2.95 ± 0.45, “perceivable” but clinically acceptable).
Ateş and Gungormus (2026)	To compare the effects of thermal aging (10,000 cycles) on color stability, surface roughness, microhardness, structural properties, and cytotoxicity of three CAD/CAM polymers: PEEK, fiber‑reinforced composite (FRC), and graphene‑reinforced PMMA (G‑CAM).	PEEK (Juvora Ltd., UK)	Fiber‑reinforced composite (Trilor, Bioloren) – glass fiber‑reinforced; Graphene‑reinforced PMMA (G‑CAM, Graphenano Dental)	Not mentioned	G‑CAM showed the lowest Δ*E*00 (best), PEEK and FRC were not significantly different from each other (both higher than G‑CAM).

**Table 4:** Summary of experimental protocols and methodological variables, including cleansing materials, staining media, aging/thermocycling processes, immersion timelines, spectrophotometer types, and finishing/polishing procedures.

First author, year	Staining medium	Cleansing materials	Other process	Timeline	Spectrophotometer	Finishing/Polishing (yes = y; no = n)
Demir Sevinç et al. (2024)	Distilled water, Cola, Red wine, Tea, Coffee, Heptane, Citric acid, 50% Ethanol	—	—	6 days, 12 days (mimic the 12‐month of daily use)	Spectrophotometer (VITA Easyshade Advance; VITA Zahnfabrik, Bad Säckingen, Germany)	y
Makkeyah et al. (2024)	Conventional cigarette smoking and heated tobacco products	—	The samples were exposed to 600 cigarettes/heets, representing 30 days of medium smoking behavior (20 cigarettes/day). Then, the samples were gently washed with distilled water for 1 min	Simulate 30 days of medium smoking behavior.	VITA Easyshade Advance 4.01 (VITA shade, VITA made, VITA)	Of the IPS e.max CAD and the Bruxzir samples, 20 samples were glazed, and 20 samples were polished, while the BioHPP samples were all polished.
Porojan et al. (2021)	Coffee, Coca‐Cola juice	—	720 cycles of thermocycling (aging)	A total of 720 cycles were used for simulating 2 min per day of contact with the respective beverages.	Vita Easyshade IV (Vita Zahnfabrik, Bad Säckingen, Germany)	y, groups: conventional polishing and glazing after polishing
Porojan et al. (2023)	Coffee, black tea, orange juice	Corega tablets, electric toothbrushing machine Oral B, Parodontax toothpaste	720 rounds of thermal cycling: about 6 h that simulates 6 months of daily 2 min exposure.	Chemical cleaning:‐ Corega tablets in 60°C water‐ 3 min/day (18 h total) Mechanical cleaning:‐ Electric brushing (Oral B, 2N pressure)‐ Parodontax toothpaste‐ 2 min/day (12 h total) Simulates 1‐year clinical use	Not mentioned	y
Shiozawa et al. (2023)	Coffee, curry, distilled water as control	—	—	Color measurements were performed after 1 day, 1 week, and 1 month of immersion.	Spectrophotometer (Crystaleye, Olympus, Tokyo, Japan)	y
Perween et al. (2025)	Distilled water, coffee, turmeric solution, Cola	—	—	Day 15 and 30: 1. Rinse (gentle distilled water). 2. Air‐dry (tissue paper). 3. Color measure. 4. Re‐immerse	Datacolor‐650 spectrophotometer	y
Polychronakis et al. (2020)	Filtered coffee, dry red wine	Corega Extradent (denture cleanser)	—	Subsequent repeated measurements of all specimens were taken at 30, 60, 90, 120, 150, 180, 210 and 240 cycles from the baseline measurement. Every cycle represented a day's effect (8 min).	Portable contact‐type colorimeter (FRU WR18; Shenzhen Wave Optoelectronics Technology Co. Ltd., Shenzhen, China)	y
Sultan and Salloum (2024)	—	—	Artificial Aging: The aging protocol included exposure to intense ultraviolet (UV) light and alternating cycles of moisture and temperature.	Not clearly mentioned	Spectrophotometer (XYZ Colorimeter, Model X1000)	Not mentioned
Abhay et al. (2021)	—	—	120,000 cycles of Chewing simulation and thermocycling	120,000 cycles of chewing simulating with thermocyclic aging	Digital spectrophotometer (Vita Easyshade Compact, Vita Zahnfabrik, Bad Säckingen, Germany)	y
Heimer et al. (2017)	Red wine, curry, chlorhexidine, distilled water	Individual PPx: Soft toothbrush, medium‐hard toothbrush, Sonic toothbrush Laboratory protocols: Sympro, SunSparkle, Ultrasonic bath, Aluminum oxide Professional PPx: Perio‐soft scaler, Sonicsys, Air Flow Comfort, Air Flow Plus	—	7 days	Portable spectrophotometer SP60 (X‐rite GmbH, MI, USA)	y
Diken Türksayar et al. (2023)	Distilled water		240,000 cycles of simultaneous mechanical (49 N, 1.2 Hz, and 0.7 mm lateral movement) and thermal aging.		VITA EasyShade V; Vita Zahnfabrik	y
Saraswathy et al. (2024)	Distilled water, black coffee, green tea, carbonated soft drink (Pepsi)	—	—	Daily 45‐min immersion for 120 days	UV‐Vis spectrophotometer (UV‐2600i, Shimadzu Corp., Nakagyo‐ku, Kyoto, Japan)	y
Papathanasiou et al. (2022)	Coca‐Cola, Coffee, Red wine, distilled water	—	—	30 days	Colorimeter (Dr Lange Microcolor Data Station; Braive Instruments)	y
Ahmed and Rattan (2023)	Tea, coffee, turmeric	—	1500 rounds of thermocycling	The measurements were carried out in the following timelines: 1, 7, and 30 days after being submerged in the staining solutions	Color reflectance spectrophotometer with computer software (SpectraMagic NX, RM2002QC, Konica Minolta Corp., Ramsey, Japan)	y
Porojan et al. (2022)	—	—	28 days of Water Saturation and 10,000 rounds of thermal cycling	Simulating 1 year of oral use.	Vita Easyshade IV (Vita Zahnfabrik, Bad Säckingen, Germany)	y
Kamal (2020)	Coffee, ginger, distilled water	—	—	7 days	Reflective spectrophotometer (Model RM200QC, X‐Rite, Neu‐Isenburg, Germany)	y
Kaya et al. (2024)	Distilled water, coffee, red wine			28 days, a storage time representing 2.5 years of clinical service	VITA EasyShade V, VITA Zahnfabrik	y
Ozyilmaz et al. (2021)	—	Corega effervescent tablet, Protefix effervescent tablet, Perlodent effervescent tablet, Curaprox citric acid‐based solution	10,000 rounds of thermal cycling	Simulation of 140 days	Spectrophotometer (VITA Easyshade, Germany)	y
Pattarakitniran et al. (2024)	Coca‐Cola, black coffee, red wine, 0.5% povidone iodine, 1% hydrogen peroxide, and distilled water	—	—	7 days (correspond to 34 to 67 months of clinical services)	Spectrophotometer (ColorQuest XE, Hunter Associates Laboratory Inc., Virginia, USA)	y
Singh et al. (2024)	—	—	The layering of both specimens with indirect composite was done using a mold with a diameter of 6 mm and a height of 2 mm + 10,000 cycles of thermocycling	10,000 cycles could approximate a year of in vivo use	Spectrophotometer CM‑5, Konica Minolta, Tokyo, Japan	Not mentioned
Sai Madhuri Nemani (2024)	—	Neutral soap solution, 10% povidone‐iodine, 4% chlorhexidine gluconate, 2% glutaraldehyde, and 5% sodium hypochlorite solution	—	30 days	Calorimeter NR200 (Spectra Labtech, Mumbai, India)	Not mentioned
Tekin (2021)	—	Corega tablet, Protefix tablet, 0.5% NaOCl solution, distilled water (control)		120 d (simulate 1 year of use)	VITA Easyshade V; VitaZahnfabrik, Bad Säckingen, Germany	y
Cinel Sahin and Mutlu Sagesen (2024)	Coffee	—	6 different polishing groups: control, “Abraso‐Starglanz” polishing paste (bredent GmbH & Co KG, Germany), “Yildiz” polishing paste (Yildiz Cila Company, Turkey), “Enhance” polishing system (Dentsply De Trey GmbH, Germany), “Super snap” polishing kit(Shofu Dental GmbH, Germany), and silicone polisher (Renfert GmbH, Germany).	30 days	Digital spectrophotometer (Vita Easy Shade V, Vita Zahnfabrik, Germany)	y
Erdağ et al. (2023)	Coffee	5% NaOCl, alkaline peroxide tablet (Corega), 0.12% chlorhexidine (Chx), toothpaste, soap, denture cleansing gel (mechanical), distilled water (control)		270 day cleaning + 7 days staining day	A digital colorimeter (Minolta CR‐321, Osaka, Japan)	y
Kar et al. (2025)	—	Listerine, Chlorhexidine (0.2%), Bioayurveda herbal mouthwash, distilled water (control)	—	180 h (simulating 15 years of daily 2‑min mouthrinse use; 12 h = 1 year equivalent)	VITA Easyshade (VITA Zahnfabrik, Germany)	y
Alharthi et al. (2025)	—	—	Thermocycling: 5000 cycles between 5°C and 55°C, 30 s dwell time (simulating 6 months of clinical service).	5000 thermal cycles (equivalent to approximately 6 months of oral temperature changes)	Agilent Technologies 5000 UV‑Vis‑NIR spectrophotometer	y
Ateş and Gungormus (2026)	—	—	Thermal cycling: 10,000 cycles (5°C–55°C, 30 s dwell, 10 s transfer)	10,000 cycles, simulated 1 year of intraoral aging	VITA Easyshade (VITA Zahnfabrik, Germany)	y

#### 3.3.1 Materials Characteristics

Across the included studies, several categories of PEEK and PEKK materials were identified. Unfilled PEEK (100% PEEK) was represented by Copra PEEK (Whitepeaks) (Demir Sevinç et al. 2024), Juvora Dental Disc (Juvora) (Ateş and Gungormus 2026; Tekin 2021; Shiozawa et al. 2023; Porojan et al. 2023), and Finoframe PEEK A2 (Fino GmbH) (Porojan et al. 2023, 2022). Ceramic‐reinforced PEEK materials were frequently examined, with BioHPP (a high‐performance polymer derived from PEEK) (Bredent) consistently described as containing 20% ceramic fillers (Porojan et al. 2021, 2023; Diken Türksayar et al. 2023; Heimer et al. 2017; Porojan et al. 2022; Abhay et al. 2021; Saraswathy et al. 2024; Makkeyah et al. 2024; Kamal 2020; Papathanasiou et al. 2022; Polychronakis et al. 2020; Alharthi et al. 2025). Pigmented and TiO_2_‐containing variants included CopraPeek Light (Whitepeaks), composed of approximately 80% PEEK and 20% titanium dioxide (Cinel Sahin and Mutlu Sagesen 2024; Erdağ et al. 2023; Sultan and Salloum 2024). PEKK materials included Pekkton Ivory (Cendres + Metaux) (Kaya et al. 2024) and another PEKK incorporating titanium dioxide pigments, from the same manufacturer (Ozyilmaz et al. 2021). Other PEEK variants included Smile PEEK (Pressing Dental) (Pattarakitniran et al. 2024) and Intamsys PEEK (Singh et al. 2024) and two unspecified PEEK CAD/CAM materials (Ahmed and Rattan R 2023; Kar et al. 2025).

#### 3.3.2 Comparisons With Other Restorative Materials

Other restorative materials were compared to PEEK in 20 studies, while only two studies used PEKK for comparison. Relative to ceramic‐based materials, PEEK frequently demonstrated reduced color stability, showing higher Δ*E* values than hybrid ceramics (Demir Sevinç et al. 2024), zirconia (Demir Sevinç et al. 2024; Abhay et al. 2021; Makkeyah et al. 2024; Kar et al. 2025), and lithium disilicate (Makkeyah et al. 2024; Kar et al. 2025). Thermocycled and worn zirconia also outperformed PEEK crowns (Abhay et al. 2021). When compared to composite resins, PEEK consistently exhibited superior color stability under exposure to staining agents such as coffee, curry, chlorhexidine, and red wine (Shiozawa et al. 2023; Heimer et al. 2017). PEEK also performed better than polymethyl methacrylate (PMMA) and acrylic resins when exposed to staining agents, artificial aging, and cleansing conditions in terms of color stability (Heimer et al. 2017; Kamal 2020; Ahmed and Rattan R 2023; Sultan and Salloum 2024; Alharthi et al. 2025). However, graphene‑reinforced PMMA (G‑CAM) showed less color change than PEEK after undergoing thermocycling (Ateş and Gungormus 2026). PEEK demonstrated lower discoloration as compared to polyamide (Valplast) (Papathanasiou et al. 2022) and a high‑performance glass fiber‑reinforced polymer (Everest C‐Temp) (Alharthi et al. 2025). Findings for acetal resin or Polyoxymethylene (POM) were inconsistent: PEEK showed less discoloration in coffee (Polychronakis et al. 2020), while acetal outperformed PEEK when exposed to coffee in another study (Kamal 2020). Direct comparisons indicated PEKK remained below the clinical acceptability threshold (Δ*E*00 < 2.25) across all staining agents (coffee and red wine), whereas PEEK did not (Kaya et al. 2024).

#### 3.3.3 Effects of Staining Agents

The type of staining medium was a major determinant for discoloration. Turmeric produced the greatest color change, frequently exceeding clinical acceptability thresholds for PEEK (Perween et al. 2025; Ahmed and Rattan R 2023). Coffee and wine also caused substantial staining for PEEK (Demir Sevinç et al. 2024; Pattarakitniran et al. 2024). Juice and black or green tea resulted in perceivable color changes (Porojan et al. 2023; Saraswathy et al. 2024; Ahmed and Rattan R 2023), while povidone–iodine caused clinically unacceptable discoloration in PEEK (Pattarakitniran et al. 2024). Cola and distilled water caused minimal discoloration compared to other staining agents (Demir Sevinç et al. 2024; Perween et al. 2025). Hydrogen peroxide mouthrinse similarly produced no significant difference (Pattarakitniran et al. 2024). Kar et al. (2025) reported that chlorhexidine and Listerine mouthrinses caused marked discoloration of PEEK, specifically in comparison to an herbal mouthwash. Among different disinfectant solutions (chlorhexidine, glutaraldehyde, povidone‐iodine, and neutral soap), sodium hypochlorite caused the most pronounced discoloration in PEEK (Chidembaranathan et al. 2024). Heated tobacco products resulted in less staining than conventional cigarette smoke; however, both were above the clinically acceptable threshold of 3.3 (Makkeyah et al. 2024). For PEKK, sodium perborate–based tablets (Perlodent) produced significant discoloration (Ozyilmaz et al. 2021), similar to PEEK, while citric acid–based cleansers led to clinically acceptable changes (Δ*E* < 3.7) in PEKK and polyamide (Ozyilmaz et al. 2021).

#### 3.3.4 Effect of Cleansers

Cleaning procedures showed variable influence on color change of PEEK. Mechanical brushing with toothpaste effectively removed visible staining from coffee and tea (Porojan et al. 2023); however, Heimer et al. (2017) emphasized that sonic toothbrushes outperform manual ones for PEEK cleaning. Denture cleansers exhibited inconsistent performance: effervescent tablets (e.g., Corega, Protefix) removed tea and coffee stains but not orange juice stains (Porojan et al. 2023). Polychronakis et al. (2020) investigated the long‐term stain removal efficacy of an alkaline peroxide–based cleanser (Corega Extradent) and found that, while this cleanser reduced discoloration by approximately 50% in POM when used in a combined coffee‐wine‐cleanser bath compared to coffee alone, it did not provide a similar protective effect for PEEK; in fact, PEEK exhibited comparable or greater discoloration in the combined bath than in coffee alone, suggesting that routine tablet use may not effectively prevent cumulative staining on PEEK over prolonged periods (Polychronakis et al. 2020). Notably, Heimer et al. (2017) reported that ultrasonic baths and Air Flow Plus are superior cleaning methods for PEEK compared to tablets or conventional brushing. Nevertheless, they cautioned that aluminum oxide air‐abrasion is contraindicated, as it damages the PEEK surface by increasing roughness above the 0.2 µm safety limit, which accelerates future staining (Heimer et al. 2017).

#### 3.3.5 Effect of Surface Treatment

Studies also showed surface finishing protocols influenced color stability outcomes for PEEK. In ceramic‐filled PEEK (BioHPP), there was no significant difference in color stability between the glazing and polishing groups after exposure to coffee and in vitro aging (Porojan et al. 2021). Polishing technique was critical: using Abraso–Starglanz paste (1 min at 3000 rpm with a polishing buff) or the Enhance Prisma Gloss system (1 min at 10,000 rpm with polishing cups and extra‐fine composite polishing paste) resulted in Δ*E* values below the clinical acceptability threshold of 1.8 after coffee immersion, whereas other protocols such as using Yildiz polishing paste, Renfert silicone polisher, and Super snap rainbow technique kit did not (Cinel Sahin and Mutlu Sagesen 2024).

#### 3.3.6 Aging and Thermal Cycling

Within restorative dentistry, aging is defined as the laboratory acceleration of time‐dependent physicochemical degradation to simulate the oral environment. Aging procedures generally had negative effects on color stability. Thermocycling adversely affected PEEK (Porojan et al. 2021), and thermomechanical aging caused ceramic‐filled PEEK to exceed the clinical acceptability threshold of 1.8 (Diken Türksayar et al. 2023). Regarding water‐induced aging, a 4‑week immersion period resulted in color changes ranging from extremely slight to slight across the evaluated PEEK materials (NBS units: 0.28–1.27). The ceramic‑reinforced BioHPP exhibited the greatest shift (NBS ≈ 1.27), whereas the unfilled Juvora demonstrated the least (NBS ≈ 0.28) (Porojan et al. 2022).

#### 3.3.7 Clinical Acceptability Thresholds

Clinical thresholds for acceptable color change (Δ*E* or Δ*E*00) varied widely among included studies. Values ranged from 1.8 to 5.6 across studies, with 3.3 being the most frequently applied threshold (Demir Sevinç et al. 2024; Heimer et al. 2017; Makkeyah et al. 2024; Pattarakitniran et al. 2024; Ahmed and Rattan R 2023; Papathanasiou et al. 2022; Alharthi et al. 2025). Other thresholds included 1.8 (Diken Türksayar et al. 2023; Cinel Sahin and Mutlu Sagesen 2024; Erdağ et al. 2023), 2.25 (Kaya et al. 2024), 2.7 (Shiozawa et al. 2023), 3.7 (Perween et al. 2025; Ozyilmaz et al. 2021), 4.08 (Tekin 2021), and 5.6 (Kamal 2020). Some studies used National Bureau of Standards (NBS) units for interpreting color changes (Porojan et al. 2021, 2022; Kar et al. 2025).

#### 3.3.8 Color Measurement Methods

Instrumental color measurement was employed in all included studies, with the exception of one study that did not report the device used (Porojan et al. 2023). The VITA Easyshade spectrophotometer (VITA Zahnfabrik) was the most frequently used device (Ateş and Gungormus 2026; Demir Sevinç et al. 2024; Tekin 2021; Porojan et al. 2021; Diken Türksayar et al. 2023; Porojan et al. 2022; Abhay et al. 2021; Makkeyah et al. 2024; Cinel Sahin and Mutlu Sagesen 2024; Kaya et al. 2024; Ozyilmaz et al. 2021; Kar et al. 2025), followed by Konica Minolta (Erdağ et al. 2023; Singh et al. 2024; Ahmed and Rattan R 2023), X‑Rite (Heimer et al. 2017; Kamal 2020), Shimadzu (Saraswathy et al. 2024), and Agilent (Alharthi et al. 2025) devices. Other devices included an Olympus spectrophotometer (Shiozawa et al. 2023), a Datacolor spectrophotometer (Perween et al. 2025), a HunterLab spectrophotometer (Pattarakitniran et al. 2024), and various colorimeters (Chidembaranathan et al. 2024). All studies measured the color parameters of the samples both before and after the exposure periods to assess color change values.

#### 3.3.9 Exposure Duration

Immersion periods ranged from 1 day to 270 days (Shiozawa et al. 2023; Cinel Sahin and Mutlu Sagesen 2024; Erdağ et al. 2023). Accelerated aging was incorporated in 11 studies using thermocycling (commonly 10,000 cycles representing 1 year) and mechanical cycling to simulate clinical service (Ateş and Gungormus 2026; Porojan et al. 2021, 2023; Diken Türksayar et al. 2023; Porojan et al. 2022; Abhay et al. 2021; Ozyilmaz et al. 2021; Singh et al. 2024; Ahmed and Rattan R 2023; Sultan and Salloum 2024; Alharthi et al. 2025).

### 3.4 Methodological Heterogeneity

Due to substantial heterogeneity across the included studies, specifically the use of more than 10 different staining agents, immersion durations ranging from 1 to 270 days, and thermocycling protocols varying from 720 to 240,000 cycles, a quantitative meta‐analysis could not be performed.

## 4 Discussion

Color stability of PEEK is critically important in the aesthetic zone, as it directly influences patient satisfaction and clinical success. This systematic review comprehensively assessed the color stability of PEEK in comparison with other commonly used dental materials under various staining and aging conditions, as well as exposure to different cleaning materials. A comparison was also made regarding the effect of the type of surface treatment and the composition of PEEK materials on their color stability. Overall, the relevant studies suggest that PEEK exhibited generally acceptable color stability; however, its performance was strongly dependent on factors such as surface treatment, staining agent, and reinforcement composition. Since there were few studies on PEKK, this systematic review mainly evaluated the color stability of PEEK.

Several studies confirmed that PEEK undergoes significantly lower color change compared with PMMA, composite resin, acetal, and polyamide (Ateş and Gungormus 2026; Heimer et al. 2017; Ahmed and Rattan R 2023; Papathanasiou et al. 2022; Polychronakis et al. 2020; Sultan and Salloum 2024). For example, PEEK exhibited substantially smaller Δ*E* values than PMMA and composite after 1 week of immersion in different staining agents (Heimer et al. 2017). Likewise, PEEK presented the lowest Δ*E* values among all tested denture base materials (BioDentaplast and polyan), supporting its potential use in removable partial denture frameworks and interim prostheses (Ahmed and Rattan R 2023). Alharthi et al. (2025) confirmed the lower discoloration of PEEK compared to PMMA; however, Ateş and Gungormus (2026) reported that graphene‑reinforced PMMA showed superior color stability as compared to PEEK. This difference was attributed to the barrier effect of graphene nanoplatelets within the graphene‑reinforced PMMA matrix, which reduces water and chromophore penetration, thereby providing better color stability than PEEK (Ateş and Gungormus 2026). Polychronakis et al. (2020) showed that acetal demonstrated markedly greater discoloration than PEEK in coffee, with a roughly fourfold increase. In contrast, Kamal (2020) reported that acetal exhibited superior color stability to PEEK following coffee exposure. This discrepancy likely arises from the different manufacturing methods (CAD/CAM milling vs. injection molding) used in two studies (Kamal 2020; Polychronakis et al. 2020). Nevertheless, upon exposure to Corega cleanser tablets and distilled water, both materials showed comparable color stability (Polychronakis et al. 2020). Moreover, as compared with PEKK, an alternative high‐performance polymer, PEEK displayed greater color change. This is because the additional ketone group in PEKK is surrounded by oxygen atoms, preventing hydrogen bonding with water molecules. This molecular difference results in lower water sorption in PEKK in comparison with PEEK and consequently greater color stability for PEKK (Kaya et al. 2024). After exposure to either cigarette smoke (Makkeyah et al. 2024) or antiseptic mouthrinses (Kar et al. 2025), PEEK consistently showed greater discoloration than ceramic materials such as zirconia and lithium disilicate, which can be attributed to the lower chemical resistance and higher susceptibility to pigment infiltration in the polymer matrix compared to densely sintered ceramic structures. Even under more aggressive mechanical aging conditions, such as thermocycling combined with chewing simulation, zirconia crowns have been shown to maintain their color significantly better than PEEK crowns (Abhay et al. 2021). Nonetheless, indirect composite veneered on PEEK frameworks demonstrated superior color stability compared with indirect composite layered on zirconia (Singh et al. 2024). This finding, in addition to the comparable bond strength between indirect resin composites and both PEEK and zirconia (Sarfaraz et al. 2020), suggests that PEEK is a suitable alternative to zirconia as a framework material in esthetic restorations (Singh et al. 2024).

Regarding the composition of PEEK materials, the extent of discoloration was critically governed by the presence or absence of reinforcing fillers. Ceramic‑reinforced PEEK (BioHPP) exhibited higher Δ*E* values than non‑reinforced PEEK following water immersion and thermocycling (Porojan et al. 2022). Specifically, water‑induced aging over a 4‑week period produced formulation‑dependent color shifts ranging from extremely slight to slight (NBS: 0.28–1.27), with ceramic‑reinforced BioHPP exhibiting the greatest alteration (NBS ≈ 1.27) and unfilled Juvora the least (NBS ≈ 0.28) (Porojan et al. 2022). Furthermore, when mechanical loading was superimposed during thermomechanical aging, ceramic‑filled PEEK exceeded the clinically acceptable Δ*E* threshold of 1.8 (Diken Türksayar et al. 2023). These findings align with the hypothesis that microstructural heterogeneity and interfacial microgaps introduced by ceramic fillers facilitate fluid sorption and pigment infiltration, thereby adversely affecting color stability. Regarding the comparison of the two fabrication methods (milling and printing), no direct study has been conducted. Nevertheless, due to the lower structural uniformity in printed PEEK materials (Limaye et al. 2022), it could be anticipated that printed PEEK specimens may be more susceptible to discoloration and staining than milled ones. This same phenomenon has been observed in 3D‑printed zirconia, where Soleimani et al. (2025) attributed the higher stainability precisely to the increased surface porosity, roughness, and lower bulk density resulting from layer fabrication. However, standard in vitro studies are warranted before making any conclusion in this regard.

Surface finishing and treatment procedures also significantly influenced the degree of discoloration in PEEK. While glazing has been shown to enhance color stability in ceramic materials such as milled and printed zirconia, zirconia‐reinforced lithium silicate, and lithium disilicate by providing a smoother, less porous surface (Soleimani et al. 2025; Kanat‐Ertürk 2020), in a study by Porojan et al. (2021). It has been demonstrated that the polished and glazed PEEK samples had no statistically significant difference in the overall color change after undergoing aging and coffee exposure (*p* = 0.061). Cinel Sahin and Mutlu Sagesen (2024) also demonstrated the effect of different polishing protocols in comparison to each other after coffee immersion. Only the “Abraso‐Starglanz” polishing paste and the “Enhance” polishing system produced Δ*E*00 values below the clinical acceptability threshold of 1.8 for PEEK, while other protocols resulted in more color change (Δ*E*00 > 1.8). Although surface roughness is often considered a factor in optical properties (Porojan et al. 2021), Cinel Sahin and Mutlu Sagesen (2024) found no significant correlation between surface roughness and color stability. Therefore, the differences in Δ*E*00 among the polishing protocols were more likely attributed to alterations in the material's absorption or adsorption capacity of the staining agent, rather than the surface roughness itself (Cinel Sahin and Mutlu Sagesen 2024). These outcomes highlight the necessity of optimized finishing procedures to maintain long‐term esthetic performance.

The type of staining solution also consistently affected the extent of discoloration for PEEK. Various studies evaluated the color stability of PEEK after exposure to green tea, coffee, Cola, Pepsi, wine, ginger, curry, turmeric, and distilled water (Demir Sevinç et al. 2024; Shiozawa et al. 2023; Porojan et al. 2021; Saraswathy et al. 2024; Perween et al. 2025; Kamal 2020; Kaya et al. 2024; Ahmed and Rattan R 2023). Green tea and turmeric produced the highest Δ*E* values compared with coffee, Pepsi, and Cola (Saraswathy et al. 2024; Perween et al. 2025). Coffee, curry, and wine also caused considerable discoloration (Shiozawa et al. 2023; Kamal 2020; Kaya et al. 2024), whereas distilled water and Cola resulted in minimal or clinically acceptable color change (Δ*E* ≤ 3.3) (Porojan et al. 2021; Perween et al. 2025; Ahmed and Rattan R 2023). These differences between the chromogenic potential of solutions may be attributed to variations in their molecular polarity and affinity for interacting with the hydrophobic and aromatic surface of PEEK (Primc 2022). Cleansing agents also directly induced color changes in PEEK, with varying degrees of severity. According to a study by Erdag et al. (2023), all tested cleaning methods, including mechanical and chemical procedures, induced clinically acceptable color change for PEEK. While Ozyilmaz et al. (2021) reported that Corega and Perlodent effervescent tablets resulted in color alterations beyond the clinically acceptable threshold. Similarly, Tekin (2021) reported that Corega tablets caused the most pronounced discoloration in PEEK among other tested denture cleansers. Among chemical cleaning agents, sodium hypochlorite produced the most severe discoloration in 30 days of exposure (Chidembaranathan et al. 2024), likely due to oxidative degradation of polymer chains. Similarly, 0.5% Povidone–iodine caused an unacceptable color change (Pattarakitniran et al. 2024), suggesting that oxidizing disinfectants should be used cautiously on PEEK restorations. Furthermore, Kar et al. (2025) reported that routine use of antiseptic mouthrinses, specifically 0.2% chlorhexidine and Listerine, caused marked discoloration of PEEK (Δ*E* up to 5.64), whereas a herbal mouthwash had a milder effect, making herbal mouthwash a suitable choice for maintaining PEEK restorations.

The effectiveness of cleaning methods varied notably depending on the type of stain and its affinity for the PEEK surface. For instance, mechanical brushing and effervescent tablets successfully removed coffee and tea stains, but they were not effective against juice‐induced discoloration (Porojan et al. 2023). Moreover, the long‐term performance of alkaline peroxide–based cleansers was not consistent across materials; although such cleansers substantially reduced staining in POM, they failed to provide a similar protective effect for PEEK, raising doubts about their routine use for preventing cumulative discoloration on PEEK (Polychronakis et al. 2020). Heimer et al. (2017) further demonstrated that ultrasonic baths and Air Flow Plus are more effective cleaning methods for PEEK than brushing or tablets; however, they cautioned against aluminum oxide air‐abrasion, as it increases surface roughness beyond the 0.2 µm threshold and consequently accelerates future staining.

### 4.1 Limitations and Future Perspectives

The considerable heterogeneity observed across studies (e.g., > 10 different staining agents, different acceptability thresholds, immersion durations 1–270 days, thermocycling 720–240,000 cycles) was a major limitation for accurate comparison of studies, and it precluded a meaningful meta‑analysis for quantitative and direct comparisons of absolute Δ*E* values between studies.

The studies included in this review exhibited considerable methodological heterogeneity regarding immersion duration, solution, and, in some cases, Δ*E* evaluation thresholds. All experiments were conducted under in vitro conditions, limiting their direct applicability to clinical environments where salivary components and oral microbiota may also influence discoloration. Further long‐term clinical research is required to evaluate the color stability of PEEK in the oral environment. Another critical limitation of the current literature is the scarcity of studies focusing on PEKK, which prevents definitive conclusions regarding its long‐term color stability.

## 5 Conclusion

Within the limitations of this systematic review on the color stability of PEEK, the following conclusions could be drawn:

1. PEEK exhibited significantly lower color change and greater color stability than conventional dental resins and polymers such as PMMA, composite, acetal, and polyamide when exposed to different staining agents and artificial aging.
2. In comparison with high‐performance ceramics (e.g., zirconia), PEEK demonstrated greater susceptibility to discoloration when not veneered by indirect composite.
3. The material composition of PEEK, particularly the addition of ceramic fillers, adversely affected its color stability.
4. Regarding surface treatment procedures, specific polishing protocols were critical for improving color stability by reducing surface roughness and pigment retention. While glazed and polished PEEKs did not show significant differences in color stability.
5. Artificial aging and thermocycling induced perceivable color changes in PEEK (Δ*E* > 0.8).
6. The type of staining solution and cleaning method significantly influenced discoloration, in which green tea, turmeric, and certain chemical cleaners (e.g., sodium hypochlorite) induced severe discoloration.
7. PEKK demonstrated better color stability than PEEK in the limited available evidence.

## Author Contributions

The corresponding author conceptualized the study, supervised the overall project, and contributed to the validation of the results and findings. The first author conducted the investigation, developed and applied the methodology, performed the majority of the data extraction and analysis, and carried out the final editing of the manuscript. The third author performed validation of the results and contributed to editing the text. All authors reviewed the manuscript, provided critical input, and approved the final version submitted for publication.

## Funding

The authors have nothing to report.

## Conflicts of Interest

The authors declare no conflicts of interest.

## Data Availability

The data that support the findings of this study are available from the corresponding author upon reasonable request.
